# Diastolic and Systolic Longitudinal Myocardial Velocities of Healthy Racing Pigeons (*Columba livia* f. domestica) Measured by Tissue Doppler Imaging

**DOI:** 10.3390/vetsci8020023

**Published:** 2021-01-31

**Authors:** Marko Legler, Lajos Koy, Norbert Kummerfeld, Michael Fehr

**Affiliations:** Clinic for Small Mammals, Reptiles and Birds, University of Veterinary Medicine Hannover, Foundation, Bünteweg 9, D-30559 Hannover, Germany; Lajos.Schmitt@gmx.de (L.K.); Nokumf@gmx.de (N.K.); Michael.Fehr@tiho-hannover.de (M.F.)

**Keywords:** TDI, tissue velocity imaging (TVI), Doppler sonography, heart, diastole, systole, birds

## Abstract

Tissue Doppler imaging (TDI) is a noninvasive sonographic method of acquiring and quantifying myocardial velocities. This technique is used in human and small animal medicine to diagnose cardiac diseases. Using this technique, we evaluated the longitudinal myocardial peak velocities of the interventricular septum, and the left and right ventricular free walls in the systole and diastole in 40 racing pigeons. The TDI examinations confirmed the movement of the heart base toward the apex in the systole and away from the apex in the diastole. Inhomogeneous distribution of the myocardial velocities with a statistically significant velocity gradient from the basal to the apical myocardial segments was found. The left and right free walls have significantly higher myocardial velocities than the myocardium of the septum. The myocardial velocities during active ventricular filling were significantly higher in the right ventricular free wall than in the left one. The validation of the method resulted in coefficients of variation between 3% and 33% for the systolic and 3% and 75% for the diastolic individual myocardial velocities. Weekly repeated measurements resulted in variation coefficients between 3% and 45% for systolic and diastolic myocardial velocities, respectively.

## 1. Introduction

Doppler sonographic techniques are used to measure and visualize blood flow in the avian heart to diagnose heart diseases [1,2,3,4,5,6,7,8,9,10,11,12,13]. Tissue Doppler imaging (TDI) or tissue velocity imaging (TVI) is a method used to quantify myocardial movement and function in human and small animal medicine [14,15,16,17,18,19]. This Doppler sonographic technique allows the quantitative measurement of the velocities of myocardial structures during the cardiac cycle. The velocity of the myocardial tissue is measured by pulsed wave tissue Doppler sampling, which shows the peak tissue velocities within a selected myocardial region over the cardiac cycle [14,17]. Studies in human and small animal medicine have worked out the prognostic role of TDI-derived parameters for cardiac diseases, such as myocardial infarction, hypertension, and heart failure [14,16,20,21,22]. Secondary heart failure due to atherosclerotic lesions is a common heart disease in avian medicine [1,2,3]. In the recent literature, blood flow velocity changes have been described in heart diseases due to atherosclerosis in parrots [3,23]. With this background, the TDI could also be a new method in avian medicine to quantify myocardial velocities and use these velocities to diagnose heart diseases. In avian medicine, this technique has not yet been used to evaluate the heart. In this study, we describe the longitudinal myocardial peak velocities in the systole and diastole measured by TDI in awake healthy racing pigeons.

## 2. Materials and Methods

The animal experiments described in this manuscript were conducted in accordance with the German animal welfare regulations and with the permission of the relevant German authorities (reference number: 33.12-42502-04-15/1864).

### 2.1. Experimental Animals

Myocardial velocities were measured in racing pigeons (*Columba livia* f. domestica; *n* = 40) of both sexes (male: *n* = 15; female: *n* = 25) at the age of 2.28 years ± 1.75 (SD) (range: 1 to 8 years) and the weight of 467.15 g ± 53.33 (SD) (range: 352–577 g). For the evaluation of body size, the sternal length of the pigeons in laterolateral radiographic images from the visible sternocoracoid joint to the end of the sternum was measured and had a characteristic size of 73.23 mm ± 3.13 (SD) (range: 63.2–80.4 mm). The pigeons were routinely trained by their owners for racing and vaccinated for pigeon avulavirus 1 (paramyxovirus 1) and salmonellosis. During the period of investigation, the pigeons were housed in indoor aviaries and were offered a commercial pigeon seed mix and fresh drinking water ad libitum. Prior to the ultrasound examination, all the pigeons showed normal drinking and feeding behavior and were acclimatized to the new aviaries for two weeks. The pigeons were tested negative for *Salmonella* and endoparasites of the intestines (composite fecal samples). Low-grade infestation with *Trichomonas gallinae* in some pigeons was treated with 10 mg of carnidazole once (Spatrix^®^, Elanco Deutschland GmbH, Bad Homburg, HE, Germany) two weeks before the beginning of the examinations. The pigeons were declared healthy by clinical, hematological, and radiological examinations. The hematocrit (44.9 ± 1.8%; 42.0–49.0%) and the buffy coat (˂ 1%) of the pigeons were within the reference values of healthy and normally hydrated pigeons [24]. The radiographically measured maximum width of the cardiac silhouette of the pigeons was within 58.7 ± 3.32% (50.3–65.1%) of the maximum width of the thorax in the range of healthy birds in the literature [1].

Female egg-laying pigeons were excluded from sonographic examinations. The influence of high abdominal pressure should be avoided in these cases.

### 2.2. Doppler Sonographic Examination

For the sonographic examination, the pigeons were fixed in a semi-upright dorsal position without anesthesia or sedation. The pigeons were held by an assistant by the neck and the extended legs with the wings were fixed against the body. The echocardiographic examinations were performed with a digital ultrasound system (Vivid 7 Dimension BT08; GE VINGMED ULTRASOUND A/S, Horten, V, Norway) equipped with a 10 MHz phased-array transducer (GE 10S-RS Probe, B Mode, 4.5–11.5 MHz; GE VINGMED ULTRASOUND A/S, Horten, V, Norway).The heart was sonographically examined from the left and right parasternal views [1] with a simultaneous electrocardiogram (ECG). The ECG was taken with clamps without teeth on the right and left patagium and the left flank fold according to Einthoven [25,26,27].

The 2D echocardiographic images were named and oriented according to Riedel (1995), Schulz (1995), and Pees et al. (2006) [1,28,29]. The right parasternal longitudinal horizontal view (“four-chamber view”) was used to measure the myocardial velocities of the left free wall, septum, and right free wall, and the right free wall was measured for the second time in the left parasternal longitudinal horizontal view. The ultrasound beam was aligned to be as parallel as possible to the direction of the myocardial longitudinal movement.

The pulsed wave TDI function of the ultrasound system was used to assess the myocardial velocities of the pigeon heart. In this software function, TDI image loops showing the velocity of the myocardial motion that is superimposed on the 2D echocardiographic images in the real-time display were recorded in connection with the ECG. The angle of view of the TDI images was set to be as small as possible to guarantee frame rates above 200 frames per second. The pulse repetition frequency value (PRF) was set to 2.5 kHz. Movements towards the transducer were color-coded in red and movements away from the transducer were color-coded in blue. The TDI images and loops were stored on the ultrasound system and evaluated offline with the Q analysis function of the ultrasound system, which is a quantitative analysis. Color-coded velocities were automatically decoded into numerical velocities for quantitative analysis with this function. Therefore, a sample area of 2 mm in diameter as a region of interest (ROI) was placed manually in the apical, middle, and basal myocardial heart wall segments of the left and right free heart walls and the septum in the four-chamber view. The myocardial velocity curves were reconstituted from the color TDI images in Q analysis without a filter setting. The myocardial systolic peak velocity (S’ wave; cm/sec), the early diastolic peak velocity (E’ wave; cm/s), and the late diastolic myocardial peak velocity (A’ wave; cm/s) were measured. The myocardial velocities of each pigeon were measured in ten sequential heart cycles and the mean was used for further evaluations after examining the distribution. Additionally, the diastolic and systolic blood flow velocities were measured by pulsed wave (PW) Doppler sonography at the level of the heart valves with a sample volume of 1.5 mm and a wall filter setting of 3.4 cm/s [9,11,30].

In six pigeons, the myocardial velocities were measured on six different days over six different weeks to calculate the weekly individual variation in the TDI measurements. The influence of the sonographic angle was also evaluated in these six pigeons. For this reason, in addition to the first measurement, the second measurement of the myocardial velocities with an increased sonographic angle over 40 degrees was performed.

### 2.3. Statistical Analysis

Statistical tests were performed using the software package SAS Enterprise 7.1. The mean, standard deviation (SD), median, and range (Xmin to Xmax) of the myocardial velocities were calculated. The Kolmogorov–Smirnov test was used to verify normal distribution. The differences between the myocardial velocities of the different heart wall segments were calculated by two-factor variance analysis (two-way ANOVA) and a subsequent Tukey–Kramer test. The influence of the ultrasound angle on the measured myocardial velocities was visualized using the Wilcoxon signed-rank test. A two-way ANOVA was used to visualize the influence of the acoustic window on the measured myocardial velocities on the right heart free wall. The day-dependent variance was calculated with a one-factor variance analysis (one-way ANOVA) and a subsequent Tukey–Kramer test. The coefficient of variation (CV) was used for the determination of the weekly and individual variance of the measurements. The percentage share of the individual and the day of examination in the error was described using an intraclass correlation coefficient. Spearman’s rank correlation coefficient was used to visualize the correlation of the heart rate and age with the TDI parameters. The significance level of *p* ≤ 0.05 was chosen (slightly significant: *p* ≤ 0.05; moderately significant: *p* ≤ 0.01; highly significant: *p* ≤ 0.001).

## 3. Results

**Figure 1:**
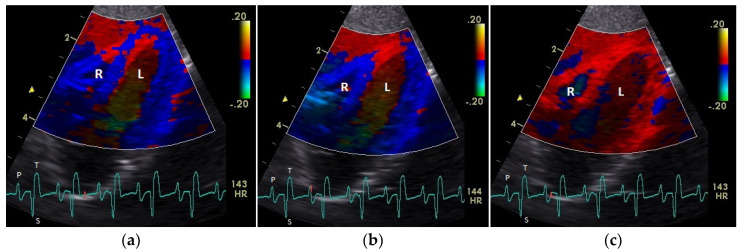
Color coding of early (**a**) and active diastolic (**b**) tissue velocities and a part of the myocardial movement during the isovolumetric contraction (**c**) by tissue Doppler imaging in the four-chamber view: the movement of the myocardium toward (red color) and away from the heart apex (blue color) is visible. R: right; L: left ventricle; HR: heart rate. Electrocardiogram: P: P wave; S: S wave; T: T wave. The color scale on the right of the image is calibrated in cm s^−1^.

**Figure 2:**
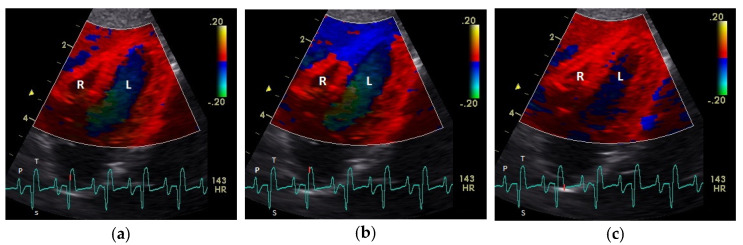
Color coding of the early (**a**), middle (**b**), and late part (c) of systolic tissue velocities by tissue Doppler imaging from the four-chamber view: the movement of the myocardium toward (red color) and away from the heart apex (blue color) is visible. R: right; L: left ventricle; HR: heart rate. Electrocardiogram: P: P wave; S: S wave; T: T wave. The color scale on the right of the image is calibrated in cm s^−1^.

The diastolic and systolic myocardial velocities could be visualized (Figure 1 and Figure 2). and measured in all the 40 examined racing pigeons (Table 1). The early (E’ wave) and late (A’ wave) diastolic velocities could be evaluated separately for all the pigeons in this study. The corresponding blood flow velocities observed in the examinations measured by PW Doppler sonography are shown in Table 2.

**Figure 3:**
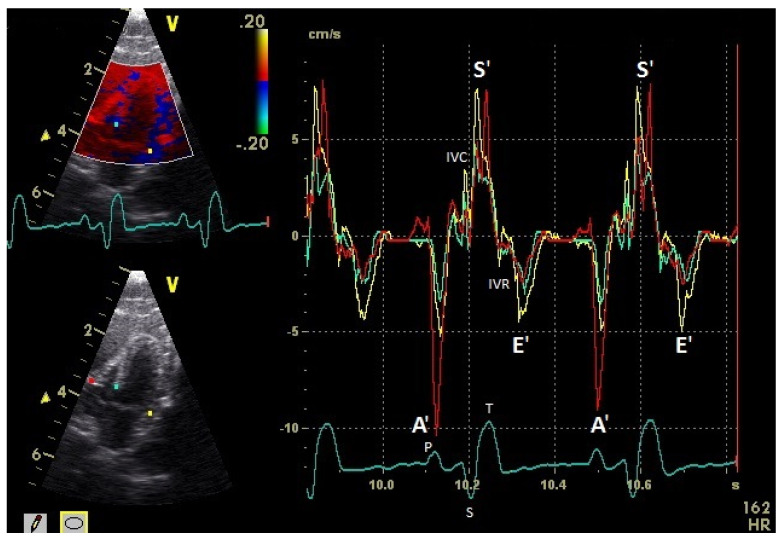
Basal myocardial velocities in the four-chamber view measured by TDI: sample areas (Ø 2 mm): red: right ventricular free wall; yellow: left ventricular free wall; green: septum; early diastolic: E’ wave; late diastolic: A’ wave; systolic tissue velocities: S’ wave. Isovolumetric contraction: IVC; isovolumetric relaxation: IVR; HR: heart rate. Electrocardiogram: P: P wave; S: S wave; T: T wave.

**Figure 4:**
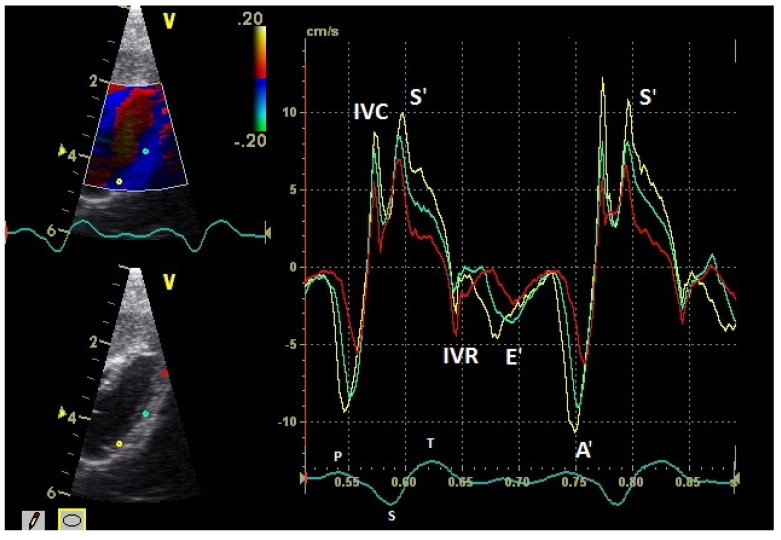
Myocardial velocities of the left ventricular free wall in the four-chamber view measured by TDI (sample areas: Ø 2 mm). The tissue velocities of the basal (yellow), middle (green), and apical (red) segment are shown: early diastolic: E’ wave; late diastolic: A’ wave; systolic tissue velocities: S’ wave. Isovolumetric contraction: IVC; isovolumetric relaxation: IVR; HR: heart rate. Electrocardiogram: P: P wave; S: S wave; T: T wave.

The positive S’ wave of the TDI curve (Figure 3 and Figure 4; ascending part of the S wave to the descending part of the T wave of the ECG) describes the systolic movement of the heart base toward the heart apex and represents the expulsion phase of the heart. In the middle of the S’ wave of the TDI, there is little movement of the heart apex toward the heart base and the myocardial velocities are negative (Figure 2). This movement in the middle part of the systole led to an irregular shape of the S’ wave in the TDI curve (Figure 3). The negative E’ wave (Figure 3 and Figure 4; after the T wave of the ECG) in the TDI curve describes the movement of the myocardium during the passive ventricular filling and the second negative A’ wave of the TDI curve (Figure 3 and Figure 4; P wave of the ECG) describes the movement of the myocardium in the active filling phases of the heart during the diastole away from the heart apex. Before and after the S’ wave, the TDI curve showed undirected deflections as a sign of isovolumetric contraction (IVC) and relaxation (IVR; Figure 3 and Figure 4). However, the transition between the A’ and S’ waves (IVC) and the S’ and E’ waves (IVR) could be flowing and sometimes difficult to see.

### 3.1. Myocardial Velocities

The systolic and diastolic peak tissue velocities depending on the myocardial segment are shown in Table 1. In general, the highest myocardial velocities are visible at the atrioventricular valvular annulus in the basal segments. The velocities decrease significantly and continuously to the apex of the heart for the S’, E’, and A’ waves (*p* ≤ 0.001). Significant differences in the myocardial velocities could also be detected between the heart septum and the left and right free wall (Table 1).

In the examined pigeons, the systolic velocities (S’ wave) of the left and right free wall were significantly higher (*p* ≤ 0.001; Table 1) than the S’ wave velocities of the heart septum. The S’ wave velocities of the right free wall were not significantly different to the velocities of the left free wall.

The diastolic E’ wave velocities (passive diastolic filling) of the left and right free wall were significantly higher than in the septum (*p* ≤ 0.001). There were no significant differences between the E’ wave velocities of the left and right free wall. The A’ wave velocities in all heart segments were higher than the E’ wave velocities (*p* ≤ 0.001). The peak A’ wave velocities of the right free wall were significantly higher than the velocities of the left free wall (*p* ≤ 0.02). The left and right free wall velocities were significantly higher than the velocities of the heart septum (*p* ≤ 0.001).

### 3.2. The Influence of Heart Rate

The heart rate was significantly correlated with the peak myocardial velocities of some heart wall segments. The myocardial velocities of the left free wall were not significantly influenced by heart rate (210.5 bpm ± 33.9 (SD); 153–274 bpm; Spearman’s rank correlation coefficient: S’ basal r = −0.08, *p* = 0.61, E’ basal r = 0.21, *p* = 0.13; A’ basal r = −0.25, *p* = 0.10). In contrast, the systolic myocardial velocities of the right free wall were significantly influenced by heart rate in the range of 130–262 bpm (200.7 bpm ± 36.7 (SD); S’ basal: *p* = 0.03, r = 0.33, middle correlation; E’ basal: *p* = 0.78, r = 0.05, no correlation; A’ basal: *p* = 0.07, r = −0.28, low correlation; Spearman’s rank correlation coefficient). The myocardial velocities of the septum were influenced significantly by heart rate (206.3 bpm ± 40.8 (SD); 138–270 bpm) only in the systole (S’ basal: *p* = 0.008, r = −0.41, middle correlation; E’ basal: *p* = 0.54, r = 0.09, no correlation; A’ basal: *p* = 0.06, r = −0.31, middle correlation; Spearman’s rank correlation coefficient).

### 3.3. Influence of Age, Sex, and Body Weight

There was no statistically significant (*p* > 0.05) influence of age, sex, or body weight on the peak myocardial velocities of the examined pigeon hearts.

### 3.4. Day-Dependent Variability in Myocardial Velocity

**Figure 5:**
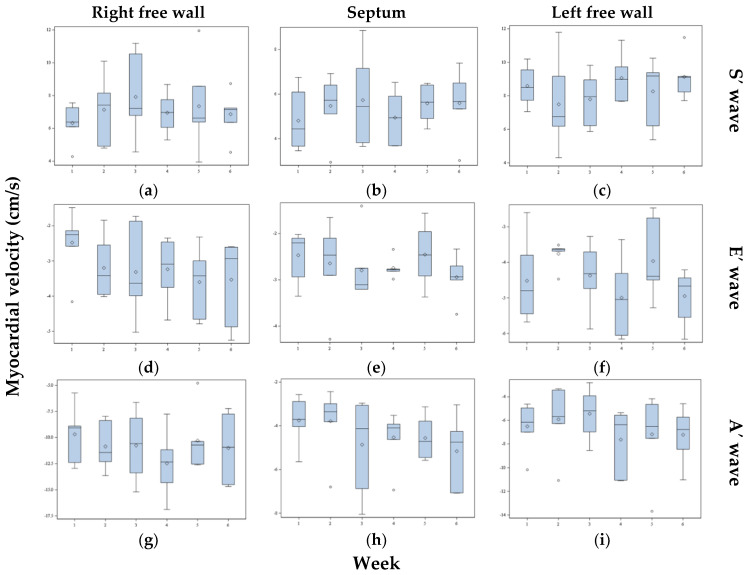
Systolic (S’ wave: **a**–**c**), early diastolic (E’ wave: **d**–**f**), and late diastolic (A’ wave: **g**–**i**) myocardial velocities of the basal heart wall segments of the right free wall, septum, and left free wall of six pigeons measured over six different weeks by TDI. The box plot shows the median (horizontal line in center), the 25th percentile (box border at bottom), and the 75th percentile (box border at top). The upper and lower ends of the whiskers show the maximum and minimum values. The single datapoints are outliers. The single diamond in the box is the mean.

Six pigeons were measured six times over six different weeks to calculate the variation range of the myocardial velocities. The measured myocardial velocities of these six pigeons are illustrated in Figure 5. There were no significant differences between the pigeons or between the examination days (*p* > 0.05). Additionally, the heart frequencies of the six pigeons were not significantly different between the six examination days (*p* > 0.05). The individual distribution of the measured values of the basal wall segments of the six pigeons was calculated by the CV shown in Table 3. The variations in the weekly measurements of the basal heart wall segments of the six pigeons are shown in Table 4. The systolic and E’ wave velocities of the septum and left free wall showed the lowest variance. The percentage distribution of the error between the individual pigeons, the observer, and the examination day is shown for the basal wall segments in Table 5.

### 3.5. Influence of the Sonographic Angle and the Sonographic Window on the Myocardial Velocities

The influence of the sonographic angle was calculated on six racing pigeons. In addition to a first measurement, a second measurement of the myocardial velocities with an increased sonographic angle of over 40 degrees was performed. The results of the basal velocities are presented in Table 6. The measurements with a greater angle deviation resulted in lower myocardial velocities. However, these findings were significant only in the middle and apical right free wall (*p* ≤ 0.41).

The influence of the sonographic window on the measured myocardial velocities of the right free wall has been reviewed. The velocities were measured from the left and right window in the examined pigeons. The results are presented in Table 1. The measured myocardial velocities of the right free wall in all heart wall segments were significantly higher in the left sonographic window (*p* ≤ 0.001). The sonographic angle was significantly lower in the left sonographic window.

## 4. Discussion

In the present study, the longitudinal myocardial peak velocities of an avian heart, the racing pigeon, were assessed for the first time. The myocardial fiber orientation in the avian heart indicates a complex ventricular motion in the systole and diastole [31,32] comparable to that of mammals and humans [33,34]. The active movement of the heart muscle is a complex twisting that consists of longitudinally and circumferentially directed movements. Additionally, there is also a passive lateral motion of the ventricular free walls during the filling and emptying of the heart chambers and a passive movement of the heart during respiration. In the literature, there is limited information about the real movement of the avian heart in the thoracic cavity. However, all these possible movements should be kept in mind when assessing the myocardial velocities. The examination of the longitudinal myocardial velocities provided a characteristic velocity distribution in the avian heart. The determined TDI curve of the examined heart segments of the pigeon heart in this study is comparable to previously described TDI curves in mammals [15,17,19] and humans [14]. In the systole, the myocardium moves in a longitudinal direction towards the apex of the heart (positive S’ wave), and during passive and active ventricular filling, the myocardium moves away from the apex of the heart (negative E’ and A’ waves). In isovolumetric contraction and dilatation, the myocardium shows positive and negative movements comparable to those of mammals [14]. These movements in particular are sometimes not easy to distinguish from S’ and E’ waves. The tissue velocities in the myocardium are non-homogeneously distributed. The typical motion of the atrioventricular valvular annulus of the heart leads in general to the highest velocities being at the basal segments with a velocity gradient to the apical segments with the lowest velocities, comparable to mammals [14,15,17,18]. The connection of the pericardium to the hepatic septum and the thoracic surface of the sternum by the sternopericardial ligament and the tight fit of the pericardium around the heart seem to be important in maintaining this heart movement with a nearly stable heart apex [31,35,36,37]. There are also differences in the velocities between the heart segments. The lowest systolic and diastolic longitudinal velocities could be found in the septum. This could be an indication of a stabilizing function of the septum in the heart’s action besides the pump function. However, the highest velocities could be detected in the left and right free walls, which is a sign of the importance of these heart segments in the pump function of the heart. The described differences between the diastolic function of the left and right ventricles [11] could also be seen in the distribution of the myocardial velocities. While the myocardial E’ wave velocities were comparable between the left and right free walls, the myocardial A’ wave velocities were significantly higher in the myocardium of the right free wall. A possible explanation for these differences is the anatomical characteristics of birds. The right ventricle lies in a crescent shape around the left ventricle; in the relaxation of the ventricles, the right ventricle is more stretched, and active ventricular filling is very important in this ventricle [31,35,36,37]. However, the relaxation of the myocardium of the left and right ventricles appears to be similar, and thus the longitudinal myocardial E’ wave velocities are equal. In this study on the myocardial velocities of pigeons, the myocardial A’ wave velocities were higher than the myocardial E’ wave velocities. This finding confirms the results of the measured blood flow velocities in pigeons, where the same results were found [11]. Thus, the E’ to A’ ratio, as well as the E to A ratio, is completely different from the mammalian one. In small animal and human medicine, the higher A wave blood flow and myocardial velocities are a sign of an increased preload in heart failure and/or ventricular relaxation abnormality [14,20,21,22]. The diastolic velocities of pigeons could indicate greater stiffness of the myocardium of birds compared to the heart muscle of mammals. A possible explanation for this could be the better transfer of the filling force of the diastole (atrial contraction) to the expulsion phase of the ventricles in the systole, especially in the higher heart rates of birds. The systolic myocardial peak velocities are similar in the left and right free walls. However, the TDI curves are slightly different. The sonographic angle in the measurements of the movements of the right heart wall is unfavorable and higher than the sonographic angle in the measurements of the left heart wall. This could mean that the velocities of the right free wall under ideal conditions could even be higher. Systolic blood flow velocities may also be higher in the pulmonary artery than in the aorta [9]. A sonographic beam-parallel alignment on the direction of the movement to be measured is recommended for the determination of velocities with echocardiography [14].

The influence of the heart rate on myocardial velocities could be demonstrated only for a few wall segments in our study. However, the heart rates in the conscious pigeons in our study should be interpreted as values at rest. The birds were trained and accustomed to handling. The heart rates were low, which can also be seen in the separated E’ and A’ as well as E and A waves. The influence of the heart rate on blood flow velocity has been previously described in the literature [8,9,11]. These tendencies could also be found in the correlation between the heart rate and the myocardial velocities. Of note is the influence on the systolic velocities of the right ventricular free wall and the diastolic A’ wave velocities of the left and right free walls. The lesser influence of the heart rate on the systolic left ventricular myocardial velocities correlates with the lesser influence of heart rate on the aortic blood flow in pigeons [9]. The influence of the heart rate on the measurements should be taken into account, especially for comparison or repeated measurements.

The age, sex, and size of the pigeons did not influence the myocardial velocities in our investigations. Nevertheless, the examined pigeons were a homogeneous group of a young age. The influence of the age on myocardial velocities is well described in humans [14] and in small animal medicine [15,38]. A possible explanation in these cases is the increasing fibrosis of the myocardium with age [14]. Whether birds show similar changes has not been sufficiently investigated. However, pathological myocardial fibrosis can occur in different heart diseases and is reported to occur in birds with atherosclerosis [39]. The influence of the breed on myocardial velocities is also well-described in dogs [15]. It can be assumed that the influence of breeds and/or species can also be found in birds, thus, in different breeds and species, specific reference values have to be established for TDI-derived parameters. The size of animals seems to play an important role in small animal medicine [15]. The first results of the examination of the myocardial velocity of parrots (macaws and grey parrots) showed similar myocardial velocities to those of pigeons [40]. In this investigation, as a result of the higher heart rates, a fused E’A’ wave had higher myocardial velocities than an isolated E’ or A’ wave. Due to the calm behavior of the animals with low heart rates in our study, the E’ and A’ waves were separated. However, in our studies on the blood flow velocities of the same pigeons with slightly higher heart rates, the E and A waves were also fused in some pigeons [11].

The determined myocardial velocities in the pigeons were strongly variable in our investigations, visible as high SD and the determined CV in repeated measurements. A similar situation can also be found in the examination of dogs and cats, with high SD and large interindividual variations [15,19]. Besides technical reasons, interindividual differences are also responsible for high variations in the myocardial velocity values in pigeons. Various physiological factors can probably be pointed to as a cause, such as heart rate or blood pressure. This makes it clear that the values of the TDI-derived parameters collected in this study should not be regarded as reference values for pigeons, because the measurements were carried out only in a small group of animals that were homogeneous in physique, training condition, and age under specified conditions. An important source of error is also the complex movement of the heart in the thoracic cavity. Over the cardiac cycle, the region of interest moves, and in every measurement, the sample area needs to be corrected. Setting an identical region in the exact cross-section plane again in repeated measurements represents a great challenge, and the non-homogenous distribution of the myocardial velocities leads to erroneous measurements. The new technique of speckle tracking can help to facilitate the tracking of a region of interest and to reduce error, and should also be evaluated in bird species [41]. In addition, a larger sonographic angle leads to a reduction in the measured longitudinal speed and also to the detection of lateral movements of the heart walls and thus to incorrect measurements. All these technical sources of error in the measurement and the individual variability of the myocardial velocities can complicate the use of myocardial velocities measured by TDI for the diagnosis of cardiac diseases. Knowledge of the differences between the TDI-derived parameters between birds with healthy hearts and birds with heart disease will be crucial for the clinical use of this technique and should be verified.

However, the advantages of the technique we used, TDI, include being able to visualize the tissue velocities in a complete sectional image plane and to measure these different myocardial velocities of different wall segments simultaneously over the cardiac cycle. TDI is an effective technique to describe heart movement and to quantify the myocardial velocities in the different heart wall segments, and thus its application for diagnosis of heart disease is possible. The anatomical characteristics of birds make the ultrasound examination of the heart, especially of the blood flow velocities in the great heart vessels, difficult. In most of these cases, the myocardium of the ventricles could be visualized and the TDI could be used to obtain information about heart function. It would be interesting to see how these myocardial velocities change in heart and vascular diseases and pericardial diseases such as pericardial effusion in pigeons and other birds. For example, the first study shows reduced myocardial velocities in advanced heart failure due to atherosclerosis and an abnormal movement of the heart apex in grey parrots with pericardial effusions [42]. However, more research is needed to evaluate myocardial velocities in diseased pigeons and other birds. In addition to its potential use in the diagnosis of heart disease, this technique can also be used to investigate the physiological heart function in birds. This can be seen, for example, in the illustration of the functioning of the right and left ventricle in our study. Furthermore, it remains to be seen how anesthesia affects myocardial velocity.

## 5. Conclusions

TDI can be used as a noninvasive sonographic method to acquire and quantify myocardial velocities in birds. The examinations of the longitudinal myocardial velocities of different heart wall segments, the septum, and the right and left free walls confirmed the movement of the heart base toward the nearly constant position of the apex in the systole and away from the apex in the diastole in pigeons. An inhomogeneous distribution of myocardial velocities with a statistically significant velocity gradient from the basal to the apical myocardial segments could be demonstrated. The left and right free walls had significantly higher myocardial velocities than the myocardium of the septum in the pigeon heart. The myocardial velocities during the active ventricular filling were significantly higher in the right ventricular free wall than in the left one, which is an indication of the different functional modes of these ventricles. The myocardial velocities of the examined pigeons were highly variable. The validation of the method resulted in coefficients of variation between 3% and 33% for the systolic and 3% and 75% for the diastolic individual myocardial velocities. Weekly repeated measurements resulted in coefficients of variation between 3% and 45% for the systolic and diastolic myocardial velocities. This variability in the measured values should be taken into account in the clinical use of the TDI.

## Figures and Tables

**Table 1:** Systolic (S’), early (E’) and late diastolic (A’) myocardial peak velocities of different heart wall segments of healthy racing pigeons measured by TDI: mean ± SD; median; range.

Myocardium	TDI	Basal (cm/s)	Middle (cm/s)	Apical (cm/s)	Heart Rate (bpm)	Sonographic Angle
Septum ^1^	S’ wave	5.4 ± 1.7; 4.7; 3.1–12.4	4.5 ± 1.3; 4.4; 2.7–8.0	3.5 ± 1.4; 3.3; 1.2–7.1	206.3 ± 40.8	18.6 ± 11.6
	E’ wave	−2.4 ± 0.6; −2.3; −1.1–−3.7	−1.9 ± 0.5; −1.8; −0.9–−3.4	−1.4 ± 0.5; −1.4; −0.4–−2.5		
	A’ wave	−3.9 ± 1.4; −3.7; −2.0–−7.4	−3.6 ± 1.4; −3.3; −1.7–−6.7	−2.7 ± 1.2; −2.5; −1.2–−5.2		
Left free wall ^1^	S’ wave	8.7 ± 1.9; 8.7; 3.7–12.3	7.4 ± 1.9; 7.2; 3.4–11.8	5.4 ± 1.9; 5.2; 1.8–9.9	210.5 ± 33.9	26.3 ± 7.5
	E’ wave	−4.4 ± 1.3; −4.3; −1.7–−7.0	−3.4 ± 1.0; −3.3; −1.4–−6.1	−2.3 ± 0.7; −2.3; −1.1–−4.0		
	A’ wave	–6.7 ± 3.0; –8.7; −2.3–−16.3	−5.9 ± 3.2; −5.1; −2.2–−18.5	−3.8 ± 2.5; −3.1; −0.6–−13.4		
Right free wall ^2^	S’ wave	8.9 ± 1.9; 8.7; 5.4–12.6	7.2 ± 1.3; 6.9; 4.2–9.6	4.8 ± 1.2; 5.0; 1.8–7.2	200.1 ± 36.7	38.9 ± 7.9
	E’ wave	−4.5 ± 1.9; −4.3; −2.3–−14.5	−3.7 ± 1.7; −3.6; −1.2–−11.5	−2.8 ± 0.8; −2.7; −1.2–−5.0		
	A’ wave	−8.8 ± 2.6; −8.7; −4.4–−12.7	−7.2 ± 2.5; −7.2; −3.7–−11.3	−4.4 ± 1.8; −4.4; −0.9–−9.6		
Right free wall ^1^	S’ wave	6.1 ± 2.11; 5.8; 2.7–12.6	5.0 ± 1.8; 4.6; 2.0–9.4	3.7 ± 1.6; 1.3; 1.3–8.2	206.9 ± 37.4	48.0 ± 11.0
	E’ wave	−2.6 ± 0.7; −2.6; −0.7–−3.8	−2.2 ± 0.8; −2.2; −0.7–−3.8	−1.7 ± 0.7; −1.7; −0.5–−3.0		
	A’ wave	−7.4 ± 2.9; –6.6; −3.2–−15.8	−5.7 ± 2.6; −4.8; −2.4–−11.8	−3.2 ± 2.2; −2.3; −0.7–−9.8		

^1^ Measured from the right sonographic window; ^2^ measured from the left sonographic window.

**Table 2:** Diastolic and systolic peak blood flow velocities (m/s) of the examined healthy racing pigeons (mean ± SD; median; Xmin–Xmax) measured by pulsed wave Doppler sonography.

Measuring Point	Early (E Wave) Diastolic Velocities	Late (A Wave) Diastolic Velocities	Systolic (S Wave) Velocities
Left atrioventricular valve ^1^	0.37 ± 0.06 (0.37; 0.25–0.53)	0.57 ± 0.20 (0.57; 0.24–0.96)	
Aorta ^2^			1.19 ± 0.16 (1.19; 0.79–1.55)
Right atrioventricular valve ^1^	0.22 ± 0.06 (0.21; 0.12–0.42)	0.53 ± 0.09 (0.52; 0.31–0.70)	
Pulmonary artery ^1^			1.08 ± 0.2 (1.06; 0.7–1.59)

^1^ Measured from the right sonographic window; ^2^ measured from the left sonographic window.

**Table 3:** Individual variation in the myocardial velocities of six pigeons measured over six weeks by TDI. The mean and standard deviation and minimal and maximal values of the coefficient of variation (CV) of each measurement are shown.

Myocardium	TDI ^1^	CV (%)
Septum	S’ wave	9.5 ± 4.7 (3–28)
	E’ wave	14.0 ± 9.6 (5–53)
	A’ wave	16.3 ± 12.0 (6–75)
Left free wall	S’ wave	7.9 ± 4.6 (2–24)
	E’ wave	14.3 ± 9.3 (4–40)
	A’ wave	13.6 ± 5.6 (3–29)
Right free wall	S’ wave	12.1 ± 5.9 (3–33)
	E’ wave	16.9 ± 8.5 (6–36)
	A’ wave	7.5 ± 3.0 (3–16)

^1^ TDI: tissue Doppler imaging.

**Table 4:** Weekly variation in the myocardial velocities of six pigeons measured six times over six weeks by TDI. The mean and standard deviation and minimal and maximal values of the coefficient of variation (CV) are shown.

Myocardium	TDI ^1^	CV (%)
Septum	S’ wave	19.2 ± 3.5 (15–24)
	E’ wave	20.0 ± 3.0 (17–25)
	A’ wave	26.8 ± 9.7 (15–41)
Left free wall	S’ wave	17.2 ± 6.1 (6–23)
	E’ wave	20.8 ± 4.7 (17–30)
	A’ wave	25.2 ± 6.0 (3–29)
Right free wall	S’ wave	21.8 ± 7.0 (10–30)
	E’ wave	25.5 ± 9.9 (17–45)
	A’ wave	21.7 ± 8.7 (12–37)

^1^ TDI: tissue Doppler imaging.

**Table 5:** The percentage distribution of the error between the individual pigeon, the observer, and the examination day of the myocardial velocities of the basal segments with an assumed error of 1 (100%) are shown.

Myocardium	TDI ^1^	Pigeon	Day	Observer
Septum	S’ wave	40.4%	45.6%	14.0%
	E’ wave	15.1%	51.7%	33.2%
	A’ wave	19.4%	47.6%	33.0%
Left free wall	S’ wave	24.3%	61.1%	14.6%
	E’ wave	6.7%	62.1%	31.2%
	A’ wave	47.9%	41.6%	10.5%
Right free wall	S’ wave	18.6%	61.0%	20.4%
	E’ wave	28.4%	47.3%	24.3%
	A’ wave	35.6%	54.3%	10.1%

^1^ TDI: tissue Doppler imaging.

**Table 6:** Basal myocardial velocities of the septum and left and right free walls depending on two different sonographic angle settings. The mean and standard deviation are shown.

Location	Sonographic Angle	S’ Wave, cm/s	E’ Wave, cm/s	A’ Wave, cm/s	Heart Rate, bpm
Septum 1	19.8 ± 6.4	4.8 ± 1.3	−2.5 ± 0.5	−3.8 ± 1.1	216 ± 50.8
Septum 2	44.5 ± 6.3	4.1 ± 0.7	−2.4 ± 0.4	−4.5 ± 1.7	243 ± 43.7
Left free wall 1	29.0 ± 3.8	8.6 ± 1.2	−4.5 ± 1.2	−6.5 ± 2.0	228 ± 46.5
Left free wall 2	39.8 ± 2.5	6.9 ± 1.7	−3.7 ± 1.4	−4.6 ± 1.2	223 ± 52.5
Right free wall 1	45.7 ± 6.2	6.3 ± 1.2	−2.5 ± 0.9	−9.7 ± 2.6	210 ± 47.4
Right free wall 2	66.8 ± 4.2	5.0 ± 2.0	−1.9 ± 0.4	−8.9 ± 3.5	225 ± 43.1

## Data Availability

The data presented in this study are available on reasonable request from the corresponding author.
